# Supplementary material for: Deletions of CDKN2A and MTAP Detected by Copy-Number Variation Array Are Associated with Loss of p16 and MTAP Protein in Pleural Mesothelioma
Source: Cancers (Basel). 2023 Oct 13;15(20):4978. doi: 10.3390/cancers15204978 (PMC10605896; doi:10.3390/cancers15204978)
Supplement: Supplementary file 1 [file cancers-15-04978-s001.zip › cancers-2634979-supplementary.pdf]

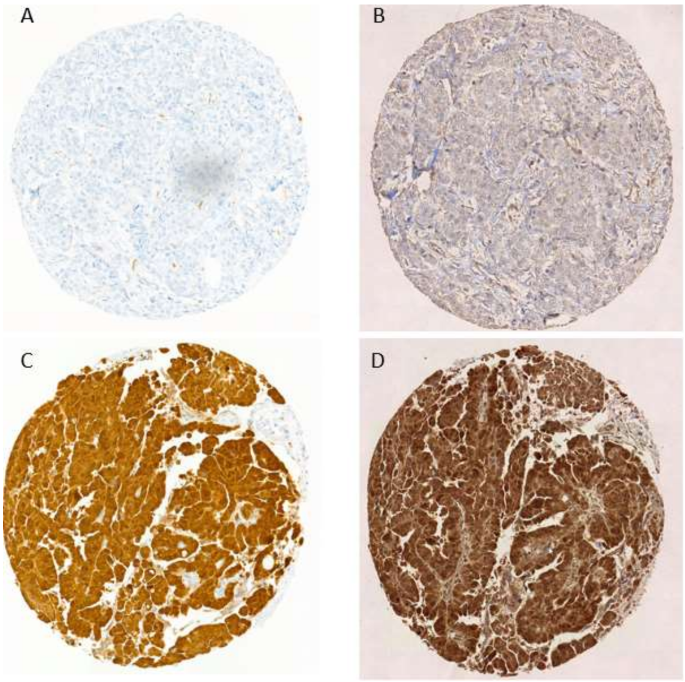

Figure S1. Negative and positive on-slide control for p16 (**A**,**C**) and MTAP (**B**,**D**) in a mammacarcinoma (**A**,**B**) and ovarian carcinoma (**C**,**D**), respectively. (Magnification 20×).
